# New Drug Expenditure by Therapeutic Area in South Korea: International Comparison and Policy Implications

**DOI:** 10.3390/healthcare13050468

**Published:** 2025-02-21

**Authors:** Seung-Rae Yu, Sooyoung Choi

**Affiliations:** 1 College of Pharmacy, Dong-Duk Women’s University, Seoul 02748, Republic of Korea; 2 Department of Biostatistics, College of Public Health and Health Professions, University of Florida, Gainesville, FL 32611, USA

**Keywords:** pharmaceutical expenditures, new chemical entities (NCEs), disease burden, DALYs, South Korea, OECD, health policy, risk-sharing agreements

## Abstract

**Background:** Pharmaceutical expenditures serve as key indicators of healthcare system efficiency, innovation, and sustainability. South Korea has implemented policies such as the economic evaluation exemption (EEE) and risk-sharing agreements (RSAs) to balance cost control and access to innovative therapies. However, discrepancies persist in the distribution of expenditures across therapeutic areas, raising concerns about alignment with public health needs. **Methods:** This retrospective observational study analyzed pharmaceutical expenditures in South Korea from 2007 to 2022, focusing on new chemical entities (NCEs). Data sources included the IQVIA MIDAS Global Database, the WHO Global Burden of Disease (GBD) database, and South Korea’s national health insurance records. Expenditure patterns were benchmarked against OECD and A8 countries using disability-adjusted life years (DALYs) and other healthcare metrics to assess the relationship between spending and disease burden. **Results:** By 2022, South Korea had introduced 276 NCEs, demonstrating progress, but still lagging the OECD average. NCE expenditure increased from 10.0% to 16.0% of total pharmaceutical spending between 2017 and 2022, whereas A8 countries’ share rose from 26.2% to 48.1%. While oncology expenditures were proportionate to disease burden, spending on chronic diseases such as musculoskeletal and cardiovascular conditions remained relatively low compared to their DALY contributions. **Conclusions:** Although South Korea has strengthened its investment in pharmaceutical innovation, disparities in expenditure distribution persist. Refining policies to enhance resource allocation for chronic diseases and expanding the RSA framework beyond oncology could improve equity and sustainability. Adopting international best practices—such as indication-based pricing and funding mechanisms for high-cost therapies—may further support optimal pharmaceutical expenditure management.

## 1. Introduction

This study is an original research article that aims to assess whether pharmaceutical spending in South Korea is appropriately aligned with disease burden, using a longitudinal approach. These expenditures reflect a system’s ability to provide timely access to effective therapies while maintaining fiscal sustainability. As healthcare systems contend with escalating costs and growing complexity, aligning pharmaceutical spending with public health objectives has become essential for sustainable and equitable outcomes [1,2]. For instance, oncology drugs represent 45% of pharmaceutical expenditures across OECD countries, despite accounting for less than 15% of the total disease burden [3]. This discrepancy arises partly from the high cost of innovative oncology treatments and the prioritization of life-extending therapies over chronic disease management. Similarly, chronic diseases such as cardiovascular and neurological disorders impose significant disease burdens, but receive disproportionately lower pharmaceutical funding, raising concerns about equitable resource allocation [4,5].

Since the implementation of national health insurance (NHI), South Korea has introduced several policies aimed at optimizing healthcare outcomes while controlling costs. Key initiatives include the economic evaluation exemption (EEE), designed to expedite access to high-cost, innovative drugs, and risk-sharing agreements (RSAs), which distribute financial risks among stakeholders. Despite these initiatives, disparities in resource allocation remain, particularly as oncology therapies continue to dominate pharmaceutical expenditures, often at the expense of chronic disease management [6,7]. While RSAs have helped mitigate financial risks associated with high-cost therapies, they have also led to budget constraints in other therapeutic areas, underscoring the need for a more holistic policy approach.

South Korea’s pharmaceutical pricing and reimbursement system follows a positive list approach, where economic evaluation plays a critical role in determining drug reimbursement decisions [8]. For general diseases, new drugs are typically assessed using a cost-effectiveness threshold of approximately 1 GDP per capita per quality-adjusted life year (QALY), while drugs for severe diseases are evaluated at a higher threshold of around 2 GDP per QALY. Within severe diseases, risk-sharing agreements are primarily used for oncology drugs, which have a relatively larger patient population, while orphan drugs for ultra-rare diseases often bypass cost-effectiveness evaluation through an economic evaluation exemption track due to challenges in demonstrating cost-effectiveness. South Korea’s RSA model predominantly follows a financial-based structure, including simple refund and expenditure cap models, which help mitigate budgetary risks while improving patient access. However, RSA coverage is currently limited to oncology and rare diseases, leading to ongoing discussions on whether this mechanism should be expanded to other high-burden chronic diseases.

Prior research emphasizes the significance of aligning healthcare spending with disease burden indicators. Chronic diseases, such as cardiovascular and neurological disorders, collectively contribute to over 30% of disability-adjusted life years (DALYs—a measure combining years lost due to premature death and disability) globally, yet they are consistently underfunded relative to their impact [6,7]. Similar trends are observed in South Korea, where oncology therapies predominate in pharmaceutical expenditures, notwithstanding the significant disease burdens posed by other conditions.

To address these disparities, this study conducts a longitudinal analysis of pharmaceutical expenditures in South Korea from 2007 to 2022. It utilizes global metrics such as DALYs from the WHO Global Burden of Disease (GBD) database and detailed expenditure data from IQVIA MIDAS, comparing South Korea’s performance with that of OECD and A8 countries [8]. This comparative analysis identifies systemic gaps in pharmaceutical expenditure alignment by benchmarking South Korea’s spending patterns against OECD and A8 countries. By leveraging longitudinal data, this study provides empirical evidence on the strengths and limitations of existing policies in achieving equitable and efficient healthcare financing.

By providing actionable insights, this study supports evidence-based policymaking to promote greater alignment between pharmaceutical spending and disease burden. The findings offer critical insights for policymakers seeking to optimize pharmaceutical resource allocation. By aligning spending priorities with disease burden, healthcare systems can achieve a more equitable balance between innovation and public health needs. These lessons are applicable beyond South Korea, informing global efforts to integrate sustainability into healthcare financing.

## 2. Methods

### 2.1. Study Design and Objectives

This study employs a retrospective observational design to examine pharmaceutical expenditures and their alignment with disease burden metrics in South Korea and other OECD and A8 countries. The primary objective is to assess the expenditure patterns of new chemical entities (NCEs) introduced from 2007 to 2022 and their alignment with public health priorities, particularly in comparison with total pharmaceutical expenditures. This study further examines expenditure distribution across therapeutic categories and reimbursement classifications, leveraging statistical methods to identify significant differences. Through this analysis, the study aims to identify policy gaps and recommend actionable strategies for equitable healthcare resource allocation.

### 2.2. Data Sources

To ensure a robust and comprehensive analysis, data were sourced from the following key resources:

1. IQVIA MIDAS Global Database: Offers detailed information on pharmaceutical sales, including expenditures and volumes, for South Korea and comparator countries [9]. Access to this database was granted through a third-party access (TPA) agreement with the Korea Research-based Pharmaceutical Industry Association (KRPIA). This facilitated data collection on pharmaceutical expenditures within public reimbursement schemes across OECD and A8 countries. To ensure data reliability, the IQVIA MIDAS dataset was cross-validated against domestic sources. This verification process involved checking the consistency of analyzed NCE lists, the study period, and therapeutic group classifications (ATC Class I–III). The comparison of MIDAS-derived expenditures with South Korea’s National Health Insurance statistics demonstrated concordance rates of 96.8% for total pharmaceutical expenditures and 94.8% for NCE-specific expenditures, supporting the validity of cross-country comparisons.
2. WHO Global Burden of Disease (GBD) data: Provides metrics on DALYs and mortality rates, categorized by disease groups and geographies, to facilitate the assessment of healthcare needs across various conditions [10].
3. South Korean national health insurance data: Contains extensive records of reimbursed pharmaceutical expenditures and ATC classification codes, accessed via the Health Insurance Review and Assessment Service (HIRA) and the National Health Insurance Service (NHIS) [11].
  ○ Total Pharmaceutical Expenditures: Data were sourced from publicly accessible platforms, including the “Annual Reports on Drug Expenditure Claims” by HIRA and statistical overviews provided by the Korean Statistical Information Service (KOSIS). These datasets underwent cross-validation with IQVIA MIDAS data to confirm their accuracy, achieving a concordance rate exceeding 95% between domestic and international datasets [12].
  ○ New Chemical Entities (NCEs): The compilation of reimbursed NCEs was obtained from the Ministry of Food and Drug Safety (MFDS) approval logs and the Ministry of Health and Welfare (MoHW) reimbursement notifications [13]. Expenditure data on NCEs were initially acquired through collaboration with the Korea Research-based Pharmaceutical Industry Association (KRPIA) and subsequently validated against data from the IQVIA MIDAS database, ensuring reliability and accuracy.
  ○ Public Data References: The annual reports on drug expenditure claims from the Health Insurance Review and Assessment Service (HIRA) [14], the statistical yearbook from the National Health Insurance Service (NHIS) [15], the National Statistics Database from the Korean Statistical Information Service (KOSIS) [16], the approval records for pharmaceuticals from the Ministry of Food and Drug Safety (MFDS) [17], and the reimbursement notifications from the Ministry of Health and Welfare (MoHW) [18].

For comparator countries (OECD and A8), all pharmaceutical expenditure and NCE-specific data were acquired solely from IQVIA MIDAS to ensure consistency and comparability in the international analyses [19]. The study’s focus was on expenditures pertaining to public reimbursement schemes, striving for comparability across ATC classifications (Class I–III) and the specified analysis periods.

### 2.3. Study Scope

The scope of the study was defined as follows:

- Geographic Scope: South Korea, 25 OECD countries (excluding South Korea), and 8 high-income A8 countries, including Canada, France, Germany, Italy, Japan, Switzerland, the United Kingdom, and the United States.
- Analysis Period: The analysis covered data from 2007 to 2022, specifically focusing on the latest six years (2017–2022) to highlight contemporary trends and patterns.
- Study Population: The study population comprised all NCEs introduced into public reimbursement schemes during the analysis period, identified based on ATC classifications and regulatory approvals of novel active substances.

### 2.4. Analytical Framework

The analytical approaches utilized to address the study objectives included the following:

- Therapeutic Categorization: NCEs were categorized according to the WHO ATC system, facilitating consistent comparisons across various countries and therapeutic domains [20].
- Key Metrics: Pharmaceutical expenditures were analyzed as a proportion of total healthcare spending and assessed with disease burden metrics such as DALYs and mortality rates [21]. To ensure consistency, all expenditure values were reported in USD (equal to KRW 1352, based on the exchange rate in the first half of 2024).
- Comparative Analysis: To provide a comprehensive analysis, two complementary approaches were applied: (1) Descriptive statistics were used to illustrate overall expenditure trends in total pharmaceutical spending [11] and NCE expenditures, and (2) Statistical hypothesis testing was performed to assess the significance of observed differences in NCE expenditure distributions across comparator groups.

### 2.5. Statistical Methods

Descriptive analyses were conducted to summarize trends in pharmaceutical expenditures, including their relative share within total healthcare spending and their alignment with disability-adjusted life years (DALYs) [10]. The cross-country comparisons used mean expenditure ratios and standard deviations to evaluate South Korea’s relative performance in pharmaceutical resource allocation. To assess differences in expenditure distribution across comparator groups, the following statistical tests were applied:

- Welch’s *t*-test: To evaluate the statistical significance of differences in NCE expenditure proportions between South Korea and the OECD average.
- Welch’s ANOVA: To analyze variations in NCE expenditure across reimbursement categories, subject to data availability constraints.

## 3. Result

### 3.1. Overview of Analyzed Pharmaceuticals

This study focused on new chemical entities (NCEs) introduced into public insurance schemes from 2007 to 2022 in South Korea and comparator countries. By 2022, South Korea had introduced 276 NCEs, while the collective OECD countries had introduced 639 NCEs. Over the past six years (2017–2022), South Korea introduced 117 NCEs, averaging 19.5 per year, compared to 313 NCEs (52.2 per year) in OECD countries. These figures highlight a distinct focus on innovative therapies in South Korea, although its NCE adoption rate remains lower than the OECD average.

### 3.2. Total Pharmaceutical Expenditures and NCE Spending

Between 2017 and 2022, total pharmaceutical expenditure in South Korea increased from USD 12 billion to USD 17 billion, representing a compound annual growth rate (CAGR) of 7.3% (Table 1). Meanwhile, pharmaceutical spending as a percentage of total healthcare expenditures declined slightly, from 25.1% in 2017 to 23.5% in 2022.

NCE expenditures in South Korea grew from USD 1.2 billion in 2017 to USD 2.7 billion in 2022, accounting for 10.0% of total pharmaceutical spending in 2017 and 16.0% in 2022. Over the same period, NCE expenditure as a proportion of total healthcare spending increased from 2.5% to 3.8%.

In OECD countries, total pharmaceutical expenditure increased from USD 2316 billion during 2017–2019 to USD 2765 billion during 2020–2022. Expenditure on NCEs rose from USD 806 billion (34.8% of total expenditures) in 2017–2019 to USD 1404 billion (50.8% of total expenditures) in 2020–2022, with a CAGR of 20.9%. Welch’s *t*-test results indicate that South Korea’s NCE expenditure share is significantly lower than the OECD mean (*p* = 0.04).

**Figure 1:**
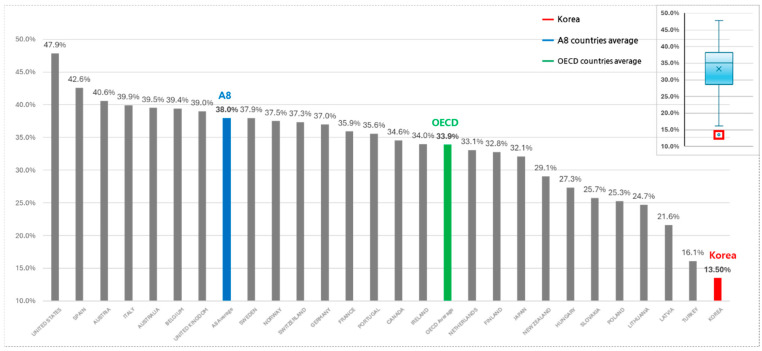
NCE expenditure share: South Korea vs. OECD and A8 countries (2017–2022). South Korea is positioned as an outlier, below the lower bound of the box plot.

When compared to A8 countries, South Korea’s share of NCE expenditure remains lower. Between 2017 and 2022, South Korea’s NCE expenditure share increased from 10.0% to 16.0%, whereas A8 countries exhibited a larger increase from 26.2% to 48.1%, with an average of 38.0% (Figure 1).

The statistical analysis confirms that Korea’s NCE/pharmaceutical expenditure ratio was significantly different from the OECD average (*p* < 0.05), particularly in recent years, as NCE investments expanded more rapidly in OECD and A8 countries.

### 3.3. WHO Burden of Disease (GBD) Analysis

#### 3.3.1. Major Causes of Death (Deaths Variable)

**Figure 2:**
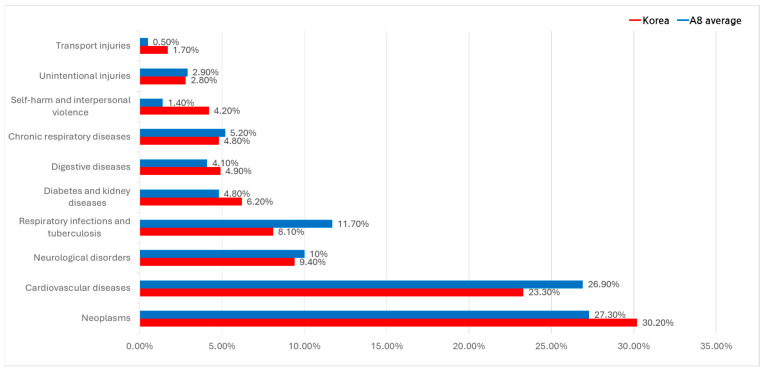
Leading causes of mortality: South Korea vs. A8 countries.

An analysis of major causes of death between 2017 and 2022, based on the Global Burden of Disease (GBD) dataset, identified malignant neoplasms (cancers) and cardiovascular diseases as the leading causes of death in both South Korea and A8 countries (Figure 2).

In South Korea, cancer accounted for 30.2% of deaths, followed by cardiovascular diseases (23.3%), neurological disorders (9.4%), respiratory infections and tuberculosis (8.1%), diabetes and kidney diseases (6.2%), and digestive diseases (4.9%).

In A8 countries, cancer (27.3%) and cardiovascular diseases (26.9%) were also the leading causes of death. Respiratory infections and tuberculosis accounted for 11.7%, followed by neurological disorders (10.0%) and chronic respiratory diseases (5.2%).

A notable difference between South Korea and A8 countries is observed in respiratory infections and tuberculosis, which accounted for a higher proportion of deaths in A8 countries (11.7%) compared to South Korea (8.1%). Additionally, chronic respiratory diseases had a higher mortality share in A8 countries (5.2%), compared to 4.0% in South Korea.

#### 3.3.2. Disability-Adjusted Life Year (DALY) Analysis

**Figure 3:**
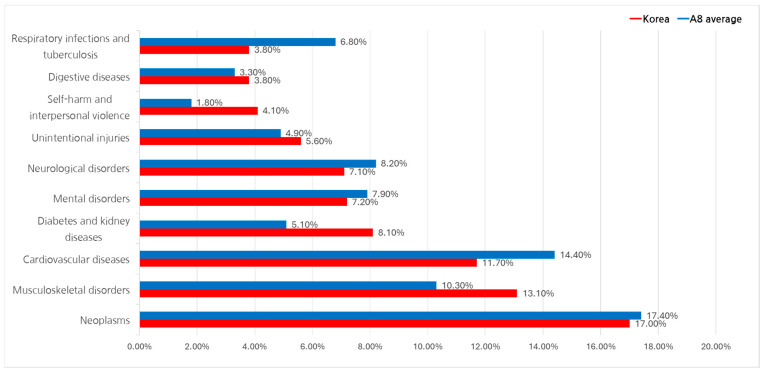
Global Burden of Disease (DALY share): South Korea vs. A8 countries.

The disability-adjusted life year (DALY) metric, developed by the WHO, combines premature mortality and years lived with disability to measure the overall burden of disease. This study utilized GBD 2021 data at Level 2 classification (22 groups) to compare disease burdens in South Korea and A8 countries (Figure 3).

Between 2017 and 2022, cancer-related diseases exhibited the highest DALY share across all regions, averaging 17.4%. In South Korea, cancer accounted for 17.0% of DALYs, followed by musculoskeletal disorders (13.1%), cardiovascular diseases (11.7%), and diabetes and kidney diseases (8.1%).

In A8 countries, cardiovascular diseases had a higher DALY share (14.4%), followed by musculoskeletal disorders (10.3%). Mental health disorders (7.9%) and neurological diseases (8.2%) also contributed significantly to the total disease burden.

### 3.4. Therapeutic Area-Specific NCE Expenditures

An analysis of therapeutic area-specific expenditures in South Korea from 2017 to 2022, based on the ATC (Anatomical Therapeutic Chemical) classification, indicates that total pharmaceutical spending reached USD 87 billion, with NCEs accounting for USD 12 billion (13.5%). Among therapeutic areas, antineoplastic agents (ATC Class L) dominated NCE spending, accounting for 46.2% of total expenditure in South Korea, followed by alimentary tract and metabolism drugs (ATC Class A, 16.0%) and genitourinary system drugs (ATC Class G, 18.3%) (Table 2). Conversely, cardiovascular system drugs (ATC Class C, 2.4%) and various pharmaceutical agents (ATC Class V, 0.2%) had the lowest shares.

In OECD countries, therapeutic area analysis revealed total pharmaceutical spending of USD 5081 billion, with USD 2210 billion allocated to NCEs, representing 33.9% of total expenditure. The highest NCE expenditure shares were observed in anti-infectives (ATC Class J, 79.2%), blood and blood-forming organs (ATC Class B, 66.3%), and antineoplastic agents (ATC Class L, 54.4%) (Table 2).

In A8 countries, NCE spending patterns were similar, with 44.3% of total pharmaceutical expenditures allocated to NCEs. The highest shares were in anti-infectives (ATC Class J, 79.1%), blood and blood-forming organs (ATC Class B, 67.9%), and antineoplastic agents (ATC Class L, 54.3%).

Statistical analysis using Welch’s *t*-test confirmed that South Korea’s NCE expenditure proportions were significantly lower than the OECD mean in key therapeutic areas (*p* < 0.05, Table 2). Specifically, the NCE spending share for cardiovascular drugs (2.4% vs. 20.2%, *p* = 0.039), blood and blood-forming organs (7.8% vs. 66.3%, *p* = 0.02), and anti-infectives (13.9% vs. 79.2%, *p* = 0.031) showed substantial differences.

**Figure 4:**
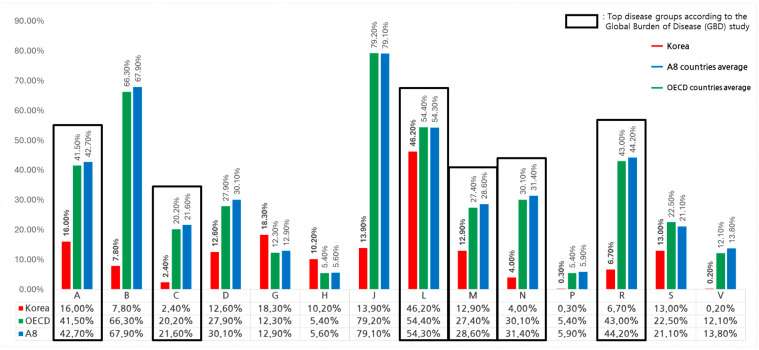
NCE spending by therapeutic area: South Korea vs. OECD and A8 countries.

A comparison across regions revealed that South Korea had the lowest NCE expenditure shares in multiple therapeutic areas, including nervous system drugs (ATC N, 4.0%) and musculoskeletal drugs (ATC M, 12.9%), relative to OECD and A8 countries (Figure 4).

#### 3.4.1. Comparative Analysis of NCE and Non-NCE Expenditures by Therapeutic Area

**Figure 5:**
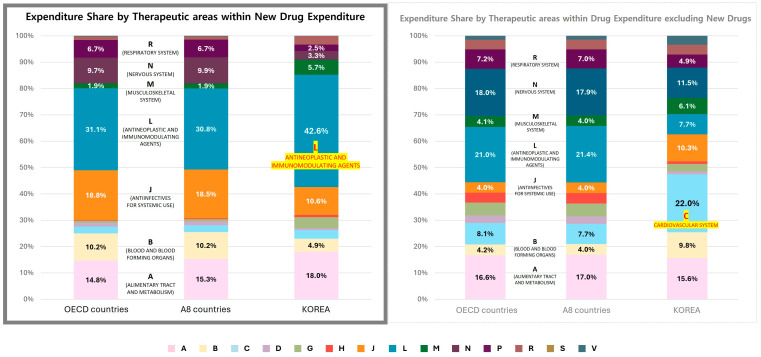
Distribution of NCE expenditure by therapeutic area. Distribution of expenditure share by therapeutic areas: (**Left**) Based on total new drug expenditure set at 100%, (**Right**) Based on total expenditure excluding new drugs set at 100%.

A comparative analysis of new chemical entity (NCE) and non-NCE expenditure across OECD countries, A8 countries, and South Korea revealed significant differences in therapeutic area spending patterns (Figure 5).

Among NCE expenditures, antineoplastic and immunomodulating agents (ATC Class L) accounted for the highest share in all regions, comprising 31.1% in OECD countries, 30.8% in A8 countries, and 42.8% in South Korea. This indicates a greater concentration of NCE spending on oncology therapies in South Korea relative to other countries.

Conversely, South Korea exhibited a lower share of NCE expenditures in anti-infectives (ATC Class J, 10.3%) compared to OECD (18.8%) and A8 countries (18.5%). Similarly, nervous system drugs (ATC Class N) accounted for only 3.0% of NCE expenditures in South Korea, significantly lower than OECD (9.7%) and A8 countries (9.9%).

For non-NCE expenditure, South Korea allocated a significantly higher proportion to cardiovascular system drugs (ATC Class C, 21.5%) compared to OECD (7.8%) and A8 countries (7.4%). This reflects a stronger emphasis on chronic disease management and essential medications in the non-NCE segment in South Korea.

While alimentary tract and metabolism drugs (ATC Class A), blood and blood-forming organs (ATC Class B), and antineoplastic agents (ATC Class L) exhibited similar NCE expenditure shares across all regions, other therapeutic categories demonstrated notable variations.

#### 3.4.2. New Drug Listing and Distribution by Efficacy Group and Drug Class

**Figure 6:**
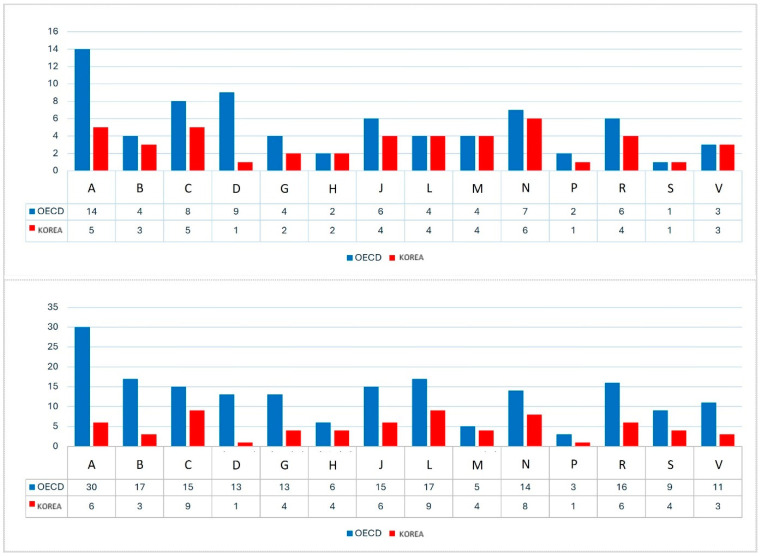
New drug listing status by efficacy group and drug class: Korea vs. OECD.

A comparative analysis of new drug listings by therapeutic area and pharmacological class between South Korea and OECD countries revealed significant differences in drug availability and spending concentration (Figure 6).

#### 3.4.3. ATC Level 2 (Efficacy Group) Analysis

At the ATC Level 2 classification (efficacy group), South Korea had NCEs listed in 45 therapeutic groups, whereas OECD countries covered 74 groups, indicating a broader distribution of new drug approvals across therapeutic areas in OECD countries.

- In South Korea, the top 10 efficacy groups accounted for 84.5% of total NCE expenditures, with antineoplastic agents (L01, 32.2%), antidiabetic drugs (A10, 12.8%), antiviral drugs (J05, 9.8%), and immunosuppressants (L04, 9.5%) leading in expenditure (Appendix A Table A1).
- In OECD countries, the top 10 groups represented 81.5% of total NCE expenditures, with antineoplastic agents (L01, 19.2%), antiviral drugs (J05, 18.4%), antidiabetic drugs (A10, 11.5%), and immunosuppressants (L04, 10.0%) ranking highest (Appendix A Table A2).
- While both regions prioritized oncology, antivirals, and diabetes drugs, OECD countries demonstrated greater diversification in drug approval across more therapeutic areas.

#### 3.4.4. ATC Level 3 (Pharmacological Class) Analysis

At the ATC Level 3 classification (pharmacological class), South Korea had NCEs listed in 68 drug classes, compared to 184 drug classes in OECD countries, suggesting a more concentrated focus on select drug classes in South Korea.

- In South Korea, the top 10 drug classes comprised 77.9% of total NCE expenditures, with protein kinase inhibitors (L01F, 15.8%), oral glucose-lowering drugs (A10B, 13.8%), and direct-acting antivirals (J05A, 9.8%) leading the list (Appendix A Table A1).
- OECD countries exhibited a more diverse spread, with antivirals for hepatitis (J5C, 11.9%), immune checkpoint inhibitors (L1G, 10.1%), and other antineoplastic agents (L1H, 6.9%) ranking highest (Appendix A Table A2).
- While South Korea showed high concentration in oncology, diabetes, and antivirals, OECD countries allocated NCE expenditures more evenly across a broader range of drug classes.

### 3.5. NCE Classification and Therapeutic Area Expenditures

Among new chemical entities (NCEs) in South Korea, the majority (58.0%) were classified under “below average weighted price” agreements, representing 51.6% of total NCE expenditure (Table 3). NCEs that underwent economic evaluation constituted 26.8% of NCEs, accounting for 41.5% of total NCE spending. Conversely, economic evaluation-exempt drugs comprised 11.6% of NCEs, representing 4.7% of total expenditure.

At the therapeutic area level, oncology (ATC Class L) had the highest number of NCEs (98), with total expenditures of USD 5014 million. Among oncology-related NCEs, those that underwent economic evaluation accounted for USD 2771 million, whereas below average weighted price drugs contributed USD 1731 million.

Conversely, musculoskeletal therapies (ATC Class M) and cardiovascular therapies (ATC Class C) exhibited lower NCE expenditures, reflecting their lower prioritization in public healthcare spending (Table 4).

Risk-sharing agreement (RSA) drugs comprised 21.4% of all NCEs, representing 28.4% of total NCE spending (Table 5). Orphan drugs, primarily used for treating rare diseases, accounted for 33.0% of NCEs, yet represented 18.2% of total expenditures. The majority of orphan drug spending was concentrated in oncology therapies (Table 6).

Statistical analysis using Welch’s ANOVA confirmed significant differences in NCE expenditures across reimbursement categories (*p* < 0.05 for multiple therapeutic areas, Table 4 and Table 6). These findings suggest that economic evaluation and pricing strategies play a critical role in shaping South Korea’s NCE expenditure distribution, with a strong emphasis on oncology and high-cost therapies.

## 4. Discussion

The findings of this study provide insights into the evolving landscape of pharmaceutical expenditures in South Korea, particularly the allocation of resources to new chemical entities (NCEs) in comparison to OECD and A8 countries. By assessing expenditure trends across therapeutic areas and reimbursement categories, this study highlights structural disparities and areas for potential policy optimization.

### 4.1. Cancer Expenditure Trends

Between 2017 and 2022, South Korea allocated 46.2% of total NCE spending to oncology drugs, significantly higher than the OECD average of 31.1% and A8 countries’ 30.8%. This reflects an increasing global trend of prioritizing cancer therapies due to their high treatment costs and clinical necessity. Similar patterns have been observed in other healthcare systems, such as Bulgaria and other European nations, where high expenditure on oncology drugs has raised concerns about sustainable financing for other disease areas [22].

While prioritizing oncology drugs is consistent with trends observed in other advanced economies, South Korea’s spending patterns suggest a relative underinvestment in chronic disease therapies, despite their substantial contribution to disease burden.

### 4.2. Underfunding of Chronic Diseases

Despite accounting for 11.7% of DALYs, cardiovascular diseases received only 2.4% of NCE spending, and nervous system drugs accounted for 4.0%, which are both considerably lower than OECD and A8 country averages. This underfunding is concerning, as musculoskeletal and cardiovascular conditions remain leading contributors to healthcare costs and productivity loss worldwide [23]. Several OECD countries have implemented policies to encourage innovation and investment in chronic disease treatments through dedicated reimbursement pathways and outcome-based pricing models [24].

South Korea’s risk-sharing agreements (RSAs) and economic evaluation exemptions (EEE) have been effective in expanding access to oncology and orphan drugs, but these policies remain largely restricted to a narrow set of therapeutic areas. Expanding these frameworks to include chronic conditions with high DALY burdens could foster a more balanced pharmaceutical expenditure distribution [25].

### 4.3. International Policy Considerations and Potential Adaptations

Managing financial risks in pharmaceutical expenditure is an increasing challenge for healthcare systems. Many OECD and A8 countries have adopted innovative pricing and reimbursement mechanisms to balance innovation incentives, cost-effectiveness, and public health priorities [26]. Several policy adaptations could be relevant for South Korea’s pharmaceutical system.

#### 4.3.1. Expansion of Risk-Sharing and Managed Entry Agreements

- Risk-sharing agreements (RSA) and managed entry agreements (MEA) are widely implemented in OECD countries to enhance access to high-cost drugs while managing financial risks [27].
- Germany and Italy use performance-based RSA models, where reimbursement is tied to demonstrated clinical effectiveness and real-world patient outcomes.
- South Korea’s RSA framework is primarily focused on oncology; broadening its application to cardiovascular and neurological diseases could improve accessibility while maintaining budget control.

#### 4.3.2. Refinement of Drug Pricing and Value-Based Assessments

- Some OECD countries, including Japan, France, and Germany, apply tiered pricing systems based on clinical benefit and innovation level [28].
- Japan considers market size, innovation level, and clinical benefit when determining drug prices, while France’s ASMR-based system classifies drugs into five reimbursement categories to differentiate funding levels.
- South Korea could adopt differential pricing strategies to ensure funding reflects therapeutic impact and long-term health outcomes.

#### 4.3.3. Funding Programs for High-Cost and Rare Disease Therapies

- Countries such as France (ATU), Germany (NUB), and the UK (CDF) have implemented dedicated funding pathways for rare diseases and high-cost treatments [29].
- South Korea could develop specialized reimbursement funds to ensure timely access to high-cost therapies while maintaining budget sustainability.

#### 4.3.4. Adaptive Pricing for Multi-Indication Drugs

- Germany, France, Italy, and Switzerland use indication-based pricing (IBP) to adjust drug costs based on real-world utilization across different indications [30].
- This approach ensures cost-effectiveness across multiple disease areas and prevents excessive spending on low-value indications.
- South Korea could assess the feasibility of IBP, particularly for oncology and immunotherapy drugs, where therapeutic impact varies across patient subgroups.

A detailed summary of international pharmaceutical pricing and reimbursement mechanisms has been compiled in Appendix A Table A3, providing a comprehensive reference for potential policy adjustments in South Korea.

### 4.4. Limitations

The findings of this study should be interpreted with the following considerations in mind. First, the study primarily analyzed pharmaceutical expenditures reimbursed under public insurance schemes, meaning that over the counter (OTC) medications and non-reimbursed treatments were not included. This limitation applies to both South Korea and comparator OECD countries, as official data on non-reimbursed drug spending remains unavailable for most healthcare systems. However, given that non-reimbursed treatments represent a relatively small share of total pharmaceutical expenditures, their exclusion is unlikely to have had a material impact on the study’s overall findings.

Second, due to differences in healthcare financing structures across countries, cross-national comparisons should be interpreted cautiously. While the IQVIA MIDAS database provides standardized expenditure data, variations in national reimbursement policies and regulatory frameworks may influence observed spending patterns. Future research could benefit from integrating additional real-world data sources, such as patient-level treatment outcomes and clinical effectiveness assessments, to refine cross-country expenditure comparisons.

Third, confidentiality agreements and data-sharing restrictions limit the availability of detailed reimbursement information in certain countries. For example, some OECD nations maintain proprietary agreements between governments and pharmaceutical manufacturers, making it difficult to obtain precise cost-sharing data for NCEs. As a result, direct comparisons of reimbursement mechanisms remain constrained by data availability.

Finally, this study does not address the long-term impact of NCE expenditures on clinical outcomes and cost-effectiveness. Future research could incorporate longitudinal analyses of patient health outcomes, drug utilization trends, and cost-effectiveness assessments to provide a more comprehensive evaluation of pharmaceutical expenditure alignment with healthcare priorities.

Despite these limitations, this study contributes to the growing body of research on pharmaceutical policy by evaluating South Korea’s NCE expenditure trends in the context of OECD and A8 countries. The findings suggest that while South Korea has made significant progress in expanding access to innovative medicines, therapeutic area disparities persist, particularly for chronic disease treatments. By adopting international best practices, including risk-sharing agreements, adaptive pricing, and dedicated funding mechanisms for high-cost therapies, South Korea could enhance the alignment between pharmaceutical spending and public health needs.

## 5. Conclusions

This study provides a comprehensive assessment of South Korea’s pharmaceutical expenditure trends, highlighting both progress and persistent disparities in resource allocation. While NCE spending has increased, particularly in oncology and orphan drug categories, significant misalignment remains for chronic diseases such as cardiovascular and neurological disorders, which account for a substantial disease burden but receive relatively lower pharmaceutical investment.

To address these gaps, South Korea could benefit from expanding risk-sharing agreements (RSAs), refining drug pricing mechanisms, and adopting value-based reimbursement models that prioritize both innovation and equitable healthcare access. International best practices, such as multi-indication drug pricing, adaptive reimbursement frameworks, and dedicated funding for high-cost treatments, offer valuable policy directions to enhance sustainability.

Future research should explore longitudinal patient outcomes, the real-world impact of NCE expenditures, and cross-country reimbursement mechanisms to further refine evidence-based pharmaceutical policies. Strengthening collaborative efforts among policymakers, healthcare providers, and industry stakeholders will be essential in achieving a balanced, cost-effective, and patient-centered healthcare system.

## Figures and Tables

**Table 1:** Comparison of total pharmaceutical and healthcare expenditure: South Korea vs. OECD.

Category	Country		2017–2019				2020–2022				CAGR	Total
			2017	2018	2019	Subtotal	2020	2021	2022	Subtotal		
Total Pharma Exp. (USD, Bil.)	Korea		12	13	14	40	15	16	17	48	7.3%	87
	OECD		734,560	771,040	810,224	2,315,825	849,471	915,501	1,000,201	2,765,173	6.4%	5,080,998
Total NCE Exp. (USD, Bil.)	Korea		1.2	1.5	1.8	4.5	2.1	2.4	2.7	7.2	17.8%	12
	OECD		215,792	267,280	322,427	805,499	385,632	461,158	557,487	1,404,276	20.9%	2,209,775
NCE/Pharma Exp. (%)	Korea		10.0%	11.4%	12.6%	11.4%	14.0%	15.3%	16.0%	15.2%	9.8%	13.5%
	OECD	Mean	23.5%	28.4%	31.8%	28.1%	34.6%	38.2%	42.6%	38.7%	13.1%	33.9%
		SD	6.4%	7.2%	6.8%	6.5%	7.4%	8.3%	9.0%	8.2%	3.6%	7.1%
	*p*-value ^†^		0.055	0.049	0.041	0.045	0.042	0.042	0.039	0.041	0.125	0.04
Healthcare Exp. (USD, Bil.)	Korea		48	54	59	161	60	65	73	198	8.7%	359
Pharma/Health Exp. (%)			25.1%	24.6%	24.1%	24.6%	24.7%	24.2%	23.5%	24.1%	−1.3%	24.3%
NCE/Health Exp. (%)			2.5%	2.8%	3.0%	2.8%	3.5%	3.7%	3.8%	3.6%	8.4%	3.3%

USD, Bil., United States dollars. Values are expressed in billions; Pharma Exp., total pharmaceutical expenditures; NCE Exp., expenditure on new chemical entities (NCEs); Healthcare Exp., total healthcare expenditure; CAGR, compound annual growth rate; Mean, average NCE/pharmaceutical expenditure (%) across OECD countries; SD, standard deviation in the NCE/pharmaceutical expenditure (%) among OECD countries, representing the variation in expenditure distribution. ^†^ *p*-value, statistical significance of differences in NCE/pharmaceutical expenditure (%) between Korea and the OECD average, calculated using an independent *t*-test (Welch’s *t*-test).

**Table 2:** Therapeutic area-specific NCE and total pharmaceutical expenditure: South Korea vs. OECD.

ATC Code ^‡^	Korea		OECD		Korea	OECD		
	Pharma Exp. (USD, Bil.)	NCE Exp. (USD, Bil.)	Pharma Exp. (USD, Bil.)	NCE Exp. (USD, Bil.)	NCE Share (%)	NCE Share (%)		
						Mean	SD	*p*-Value ^†^
A	13	2.2	785	326	16.0%	41.5%	7.6%	0.035
B	8.0	0.6	341	226	7.8%	66.3%	10.0%	0.02
C	17	0.4	281	57	2.4%	20.2%	6.0%	0.039
D	0.8	0.1	111	31	12.6%	27.9%	9.6%	0.073
G	2.6	0.5	151	19	18.3%	12.3%	7.1%	0.135
H	0.7	0.1	112	6.1	10.2%	5.4%	2.4%	0.058
J	8.7	1.2	526	416	13.9%	79.2%	17.3%	0.031
L	11	5.0	1266	688	46.2%	54.4%	4.0%	0.057
M	5.1	0.7	154	42	12.9%	27.4%	5.4%	0.043
N	8.7	0.3	710	214	4.0%	30.1%	7.1%	0.032
P	0.04	0.0001	6.5	0.3	0.3%	5.4%	2.7%	0.061
R	3.8	0.3	347	149	6.7%	43.0%	14.7%	0.047
S	3.1	0.4	129	29	13.0%	22.5%	12.0%	0.144
V	2.5	0.0	48	5.9	0.2%	12.1%	6.4%	0.062
Total	87	12	5081	2210	13.5%	33.9%	7.1%	0.04

USD, Bil., United States dollars. Values are expressed in billions; Pharma Exp., total pharmaceutical expenditures; NCE Exp., expenditure on new chemical entities (NCEs); NCE Share (%), proportion of NCE expenditure relative to total pharmaceutical expenditures; Mean, average NCE share (%) across OECD countries; SD, standard deviation in the NCE share (%) among OECD countries, representing the variation in expenditure distribution. ^†^ *p*-value, statistical significance of differences in NCE/pharmaceutical expenditure (%) between Korea and the OECD average, calculated using an independent *t*-test (Welch’s *t*-test). ^‡^ ATC Code, Anatomical Therapeutic Chemical (ATC) classification system for categorizing pharmaceuticals by therapeutic use; A, alimentary; B, blood and hematopoietic; C, cardiovascular; D, dermatological; G, genitourinary; H, endocrine system; J, anti-infectives; L, antineoplastics; M, musculoskeletal; N, nervous system; P, antiparasitics; R, respiratory; S, sensory organs; V, various.

**Table 3:** NCE expenditure by reimbursement category in South Korea.

Category	Economic Evaluation Performed	Economic Evaluation Exempt	Below Avg. Weighted Price	Clinically Essential Drugs	Total
NCE Count	74	32	160	10	276
Share in NCE Count (%)	26.8%	11.6%	58.0%	3.6%	100%
NCE Exp. (USD, Mil.)	4868	547	6053	255	11,723
Share in NCE Exp. (%)	41.5%	4.7%	51.6%	2.2%	100%
Share in Total Pharma Exp. (%)	5.6%	0.6%	6.9%	0.3%	13.50%

USD, Mil., United States dollars. Values are expressed in millions; NCE Count, number of NCEs according to reimbursement classification type; NCE Exp., expenditure on NCEs categorized by reimbursement classification; Total Pharma Exp., total pharmaceutical expenditures; Below Avg. Weighted Price, drugs reimbursed below the average weighted price; Clinically Essential Drugs, drugs deemed essential for clinical treatment.

**Table 4:** NCE expenditure by reimbursement category and therapeutic area in South Korea.

ATC Code ^‡^	Economic Evaluation Performed (USD, Mil.)				Economic Evaluation Exempt (USD, Mil.)				Below Avg. Weighted Price (USD, Mil.)				Clinically Essential Drugs (USD, Mil.)				*p*-Value ^†^
	N	Sum	Mean	SD	N	Sum	Mean	SD	N	Sum	Mean	SD	N	Sum	Mean	SD	
A	7	633	9.3	11	3	58	3.9	4.0	28	1421	8.4	14	4	47	1.8	2.9	0.01
B	6	202	3.1	2.8	1	24	4.0	1.6	5	380	8.6	10	1	16	1.2	1.2	<0.001
C	4	61	1.7	1.7		-	-	-	10	339	2.8	4.8		-	-	-	0.039
D	1	107	36	18		-	-	-		-	-	-		-	-	-	N/A
G	1	119	4.9	3.4		-	-	-	6	350	5.3	10		-	-	-	0.074
H	1	36	6.0	1.0		-	-	-	5	39	0.9	1.3		-	-	-	<0.001
J	7	212	5.0	12	1	0.6	0.6	-	25	1001	8.9	21		-	-	-	0.858
L	34	2771	9.4	15	22	333	3.0	4	40	1731	5.1	8.7	2	179	8.5	9.2	0.001
M	1	294	49	38	2	124	18	11	8	237	5.8	5.8		-	-	-	<0.001
N	5	81	1.0	1.1	1	6	1.1	0.6	16	250	1.6	2.2	2	13	0.8	1.5	0.057
P		-	-	-		-	-	-	1	0.1	0.0	0.0		-	-	-	N/A
R	2	4.9	0.5	0.4		-	-	-	10	250	3.1	4.2		-	-	-	<0.001
S	4	345	14	20		-	-	-	5	56	2.0	2.1		-	-	-	0.003
V	1	3.4	1.1	0.5	2	1.5	0.3	0.6	1	0.04	0.04	-	1	-	-	-	<0.001
Total	74	4868	7.3	14	32	547	3.6	5.6	160	6053	5.0	11	10	255	3.3	6.0	0.107

USD, Mil., United States dollars. Values are expressed in millions; N, number of NCEs in each reimbursement category; Sum, total NCE expenditure in each category; Mean, average expenditure per NCE; SD, standard deviation of NCE expenditure in each category; Economic Evaluation Performed, NCEs that underwent economic evaluation as part of the reimbursement process; Economic Evaluation Exempt, NCEs exempt from economic evaluation; Below Avg. Weighted Price, NCEs reimbursed below the average weighted price; Clinically Essential Drugs, NCEs deemed essential for clinical treatment. ^†^ *p*-value, statistical significance of differences in NCE expenditure across reimbursement categories, calculated using Welch’s *t*-test or Welch’s ANOVA (N/A, insufficient data for comparison). ^‡^ ATC Code, Anatomical Therapeutic Chemical (ATC) classification system for categorizing pharmaceuticals by therapeutic use; A, alimentary; B, blood and hematopoietic; C, cardiovascular; D, dermatological; G, genitourinary; H, endocrine system; J, anti-infectives; L, antineoplastics; M, musculoskeletal; N, nervous system; P, antiparasitics; R, respiratory; S, sensory organs; V, various.

**Table 5:** NCE expenditure by RSA inclusion and orphan drug status in South Korea.

Category	RSA Inclusion		Orphan Drug Designation		Total
	RSA Included	RSA Excluded	Orphan Drugs	Non-Orphan Drugs	
NCE Count	59	217	91	185	276
Share in NCE Count (%)	21.40%	78.60%	33.00%	67.00%	100%
NCE Exp. (USD, Mil.)	4504	11,346	2890	12,960	15,850
Share in NCE Exp. (%)	28.4%	71.6%	18.2%	81.8%	100%
Share in Total Pharma Exp. (%)	3.8%	9.6%	2.5%	11.0%	13.5%

USD, Mil., United States dollars. Values are expressed in millions; NCE Count, number of NCEs classified by RSA and orphan drug status; NCE Exp., expenditure on NCEs categorized by RSA and orphan drug status; Total Pharma Exp., total pharmaceutical expenditures; RSA, risk-sharing agreements, reimbursement contracts that include conditional coverage or cost-sharing mechanisms for high-cost drugs; RSA Included, NCEs covered under an RSA; RSA Excluded, NCEs without an RSA; Orphan Drugs, NCEs designated for treating rare diseases; Non-Orphan Drugs, NCEs without orphan drug designation.

**Table 6:** NCE expenditure by RSA inclusion and orphan drug status by therapeutic area in South Korea.

ATC Code ^‡^	RSA Inclusion (USD, Mil.)								Orphan Drug Designation (USD, Mil.)								*p*-Value ^†^
	RSA Included				RSA Excluded				Orphan Drugs				Non-Orphan Drugs				
	N	Sum	Mean	SD	N	Sum	Mean	SD	N	Sum	Mean	SD	N	Sum	Mean	SD	
A	3	58	3.9	4.0	39	2101	8.0	13	14	219	2.8	4.4	28	1941	10	14	0.015
B	2	31	1.5	1.9	11	591	5.5	7.4	7	76	1.2	1.4	6	546	8.0	8.3	<0.001
C		-	-	-	14	399	2.6	4.3	4	34	1.2	0.8	10	365	2.9	4.7	0.006
D	1	107	36	18		-	-	-		-	-	-	1	107	36	18	0.052
G		-	-	-	7	469	5.2	8.7		-	-	-	7	469	5.2	8.7	0.763
H		-	-	-	6	76	1.6	2.1	3	10	0.4	0.7	3	66	2.6	2.3	0.001
J	1	0.6	0.6	-	32	1212	7.9	19	4	49	2.4	2.7	29	1163	8.7	21	0.011
L	47	3004	9.1	15	51	2010	4.6	7.0	49	1586	5	11	49	3428	7.7	12	0.739
M	2	124	18	11	9	531	11	20	3	143	12	11	8	512	12	21	0.145
N	1	6	1.1	0.6	23	344	1.3	1.9	4	19	0.8	1.3	20	330	1.4	1.9	0.089
P		-	-	-	1	0.1	0.02	0.02		-	-	-	1	0	0.02	0.02	0.937
R		-	-	-	12	255	2.8	4.0			-	-	12	255	2.8	4.0	0.324
S		-	-	-	9	401	7.7	15		-	-	-	9	401	7.7	15	0.259
V	2	1.5	0.3	0.6	3	3	0.9	0.7	3	1.5	0.3	0.6	2	3	0.9	0.7	<0.001
Total	59	3331	8.6	15	217	8392	4.9	10.3	91	2137	3.7	8.5	185	9586	6.3	12	0.006

USD, Mil., United States dollars. Values are expressed in millions; N, number of NCEs classified by RSA and orphan drug status; Sum, total NCE expenditure in each category; Mean, average expenditure per NCE; SD, standard deviation of NCE expenditure in each category; RSA, risk-sharing agreements, reimbursement contracts that include conditional coverage or cost-sharing mechanisms for high-cost drugs; RSA Included, NCEs covered under an RSA; RSA Excluded, NCEs without an RSA; Orphan Drugs, NCEs designated for treating rare diseases; Non-Orphan Drugs, NCEs without orphan drug designation. ^†^ *p*-value, statistical significance of differences in NCE expenditure across reimbursement categories, calculated using Welch’s *t*-test or Welch’s ANOVA. ^‡^ ATC Code, Anatomical Therapeutic Chemical (ATC) classification system for categorizing pharmaceuticals by therapeutic use; A, alimentary; B, blood and hematopoietic; C, cardiovascular; D, dermatological; G, genitourinary; H, endocrine system; J, anti-infectives; L, antineoplastics; M, musculoskeletal; N, nervous system; P, antiparasitics; R, respiratory; S, sensory organs; V, various.

## Data Availability

Data are contained within the article.

## Appendix A

**Table A1:** New drug expenditure by detailed efficacy and pharmacological classification in South Korea.

ATC Code		ATC Definition	New Drug Expenditure (USD, Mil.)	Share of New Drugs in Total Expenditure
ATC level 2	L01	Antineoplastic agents	3780	32.2%
	A10	Antidiabetic drugs	1503	12.8%
	J05	Antiviral drugs	1145	9.8%
	L04	Immunosuppressants	1112	9.5%
	B01	Antithrombotic agents	586	5.0%
	G04	Urologicals	409	3.5%
	A02	Drugs for acid-related disorders	405	3.5%
	S01	Ophthalmologicals	401	3.4%
	M05	Drugs for bone diseases	320	2.7%
	C09	Agents acting on the renin–angiotensin system	249	2.1%
	Other 35 ATC Level 2 Groups		1815	15.7%
	Total		11,723	100%
ATC level 3	L01F	Protein kinase inhibitors	1855	15.8%
	L01E	Blood glucose-lowering drugs, excluding insulins	1613	13.8%
	A10B	Blood glucose-lowering drugs, excluding insulins	1374	11.7%
	J05A	Direct acting antivirals	1145	9.8%
	L04A	Immunosuppressants	1112	9.5%
	B01A	Antithrombotic agents	586	5.0%
	A02B	Peptic ulcer and gastro-oesophageal reflux disease	405	3.5%
	G04B	Drugs used in benign prostatic hyperplasia	376	3.2%
	S01L	Ophthalmologicals, antineovascularisation agents	345	2.9%
	M05B	Drugs Affecting bone structure and mineralization	320	2.7%
	Other 58 ATC Level 3 Groups		2594	22.1%
	Total		11,723	100%

USD, Mil., United States Dollars. Values are expressed in millions; New Drug Expenditure, total expenditure on new chemical entities (NCEs) in each therapeutic category; Share of New Drugs in Total Expenditure, proportion of NCE expenditure relative to total pharmaceutical expenditures.

**Table A2:** New drug expenditure by detailed efficacy and pharmacological classification in OECD countries.

ATC Code		ATC Definition	New Drug Expenditure (USD, Bil.)	Share of New Drugs in Total Expenditure
ATC level 2	L01	Antineoplastic agents	424	19.2%
	J05	Antiviral drugs	407	18.4%
	A10	Antidiabetic drugs	255	11.5%
	L04	Immunosuppressants	221	10.0%
	B01	Antithrombotic agents	171	7.8%
	N07	Other nervous system drugs	89	4.0%
	R03	Drugs for respiratory diseases	82	3.7%
	R07	Other respiratory system drugs	66	3.0%
	N05	Psycholeptics	45	2.0%
	L02	Endocrine system drugs	40	1.8%
	Other 67 ATC Level 2 Groups		408	18.5%
	Total		2209	100%
ATC level 3	J5C	Antivirals for hepatitis	263	11.9%
	L1G	Immune checkpoint inhibitors	222	10.1%
	L1H	Other antineoplastic agents	152	6.9%
	B1F	Thrombolytics	151	6.8%
	L4C	Selective immunosuppressants	133	6.0%
	J5D	Antivirals for HIV	133	6.0%
	A10S	Sodium–glucose co-transporter 2 (SGLT2) inhibitors	124	5.6%
	A10P	Combination of oral blood glucose-lowering drugs	83	3.8%
	N7A	Drugs used in addictive disorders	63	2.9%
	L4X	Other immunosuppressants	58	2.6%
	Other 174 ATC Level 3 Groups		826	37.4%
	Total		2209	100%

USD, Bil., United States dollars. Values are expressed in billions; New Drug Expenditure, total expenditure on new chemical entities (NCEs) in each therapeutic category; Share of New Drugs in total expenditure, proportion of NCE expenditure relative to total pharmaceutical expenditures.

**Table A3:** International pricing systems for reflecting new drug value and enhancing patient access.

Policy Type	Country	Key Features
Management of Therapeutic Outcomes and Financial Expenditure Uncertainty	Multiple Countries	Common policies include risk-sharing agreements (RSAs), patient access schemes, confidential commercial agreements, and managed access agreements. These models employ performance-based, financial-based, or mixed frameworks, as well as early reimbursement with post-evaluation mechanisms.
Refinement of Clinical/Therapeutic Value Assessment and Pricing Incentives	Japan	Drug evaluation considers multiple factors such as indication, market environment, innovation, utility, pediatric use, and priority introduction, incorporating various pricing incentives.
	Switzerland	Innovative new drugs receive a price premium of up to 20% for a maximum of 15 years compared to existing drugs.
	France	The clinical utility of drugs is assessed through absolute therapeutic benefit (SMR) and relative improvement in benefit (ASMR), classified into five tiers. This affects reimbursement rates, fast-track approvals, and post-market price adjustments, including price reductions and rebates.
	Germany	Six-tiered evaluation system for innovative drugs, which influences pricing decisions.
	Italy	Drugs are categorized into three tiers based on their level of innovation, and this classification impacts price negotiations.
	Belgium	Drugs are assessed based on therapeutic value, with an additional benefit premium applied.
	Canada	A four-tier system evaluates therapeutic improvements, determining the maximum allowable price.
	Taiwan	A three-tier classification evaluates clinical value improvement, with special pricing mechanisms for innovative drugs and orphan drugs without alternatives.
Dedicated Financial Support for Severe Disease Treatments and Patient Access Programs	France	Temporary use authorization (ATU) allows pre-approval use of drugs for severe and rare diseases.
	Germany	New Technologies and Unmet Needs Fund (NUB) supports rare disease treatments, oncology drugs, and novel diagnostic/therapeutic methods.
	Italy	AIFA fund supports the reimbursement and research and development of orphan drugs.
	United Kingdom	Separate funds for non-recommended oncology drugs (CDF) and rare disease treatments (IMF) ensure patient access.
	Scotland	New Medicines Fund (NMF) provides financial support for rare, ultra-rare, and end-of-life treatments.
	Australia	Life-Saving Drugs Program (LSDP) supports high-cost treatments for life-threatening and rare diseases.
	Belgium	Special Solidarity Fund (SSF) provides separate financial support for high-cost drugs for severe diseases not covered by national health insurance.
	Canada	Special Access Program (SAP) grants access to unauthorized drugs for life-threatening and severe diseases. Some provinces have additional specialized programs: RDDCP (Alberta), OPDP (Ontario), and SDP.
	New Zealand	Compassionate Fund (CF) provides financial assistance for rare and difficult-to-treat diseases.
Relaxation or Exemption of Economic Evaluation Criteria	United Kingdom	Highly Specialized Technology (HST) assessment raises the ICER threshold, considering drug characteristics, clinical utility, willingness to pay, and social demands.
	Germany	Orphan drugs and treatments below a certain financial threshold are exempt from submitting a full cost–benefit report.
	Australia	Rule of Rescue allows reimbursement for essential drugs even if cost-effectiveness thresholds are not met.
Diversification of Drug Payment Programs	United States	For high-cost drugs, pharmaceutical companies may split payments to insurers or allocate different insurance coverage per disease, spreading financial burdens.
Multi-Indication Drug Value Assessment and Pricing Adjustments	Germany	When a new indication is added, the G-BA sets a comparative group for each indication, evaluating additional benefits and target population size, leading to weighted average pricing based on usage.
	France	The drug pricing committee sets a single weighted average price, considering clinical equivalence, expected usage, and therapeutic value for each indication.
	Italy	Managed entry agreements (MEAs) apply different rebate rates per indication, with mandatory patient registries for monitoring.
	Australia	Selective price adjustment (SPA) applies differentiated reimbursement rates for drugs demonstrating substantial benefits over alternatives in severe diseases and specific patient groups.
	United Kingdom	Clinical benefit and unmet needs for each indication determine separate reimbursement rates, primarily applied to targeted therapies and immuno-oncology drugs.
	Switzerland	Reimbursement codes are assigned per indication, enabling indication-based price adjustments and usage monitoring.
Incentives for Innovative Drugs	Italy	The Innovative Drug Fund (IDF) provides a 10% price premium for selected drugs.
Specialized Reimbursement Programs	United States	The Specialty Pharmacy Program offers personalized services for rare disease treatments, requiring additional enrollment for coverage.
